# Bronchial Asthma With Scimitar Syndrome: A Case Report

**DOI:** 10.7759/cureus.51823

**Published:** 2024-01-07

**Authors:** Syed F Jamil, Elham H Alsalahi, Abaad A Alamri, Majd A Alsaman

**Affiliations:** 1 College of Medicine, King Saud Bin Abdulaziz University for Health Sciences, Riyadh, SAU; 2 Research, King Abdullah International Medical Research Center, Riyadh, SAU; 3 Pedaitrics, King Abdullah Specialized Children's Hospital, Riyadh, SAU

**Keywords:** congenital pulmonary artery hypoplasia, dextrocardia, chest infection, lung agenesis, infantile scimitar syndrome

## Abstract

Scimitar syndrome is a rare congenital cardiopulmonary anomaly; it is also called venolobar syndrome, hypogenic lung syndrome, and Halasz syndrome. The syndrome is characterized by cardiac dextroposition, right lung and pulmonary artery hypoplasia as well as complete or partial anomalous pulmonary venous drainage of the right lung. We report a case of a 22-month-old full-term male child with a severe form of scimitar syndrome diagnosed at birth. The X-ray demonstrated dextrocardia and right lung hypoplasia, while the echocardiography clearly illustrated the scimitar vein. The patient had multiple ER visits and hospitalizations due to asthma exacerbation that was aggravated by recurrent respiratory tract infections; he responded well to asthma medications during his admissions yet compliance to his prophylactic asthma medications was poor at home.

## Introduction

Scimitar syndrome was first described in 1836, and its incidence is estimated to be around 1-3/100,000 live births [1]. It is a rare congenital cardiopulmonary anomaly characterized by partial anomalous venous drainage of the right pulmonary artery to the inferior vena cava rather than the right atrium, hypoplasia of the right lung and pulmonary artery, and dextroposition of the heart. It is often associated with ostium secundum atrial septal defect (ASD), patent ductus arteriosus (PDA), or, less commonly, ventricular septal defects (VSD). Its incidence is noted to be higher among females compared to males, with a female-to-male ratio of 2:1 [1-2]. The name of the syndrome refers to the classical radiological finding of the abnormal draining vein shaped like a curved Turkish sword (scimitar), with a tubular structure running parallel to the right cardiac border [3]. The most important complications associated with the syndrome are recurrent pulmonary infections, congestive heart failure, and pulmonary hypertension [4].

## Case presentation

**Figure 1:**
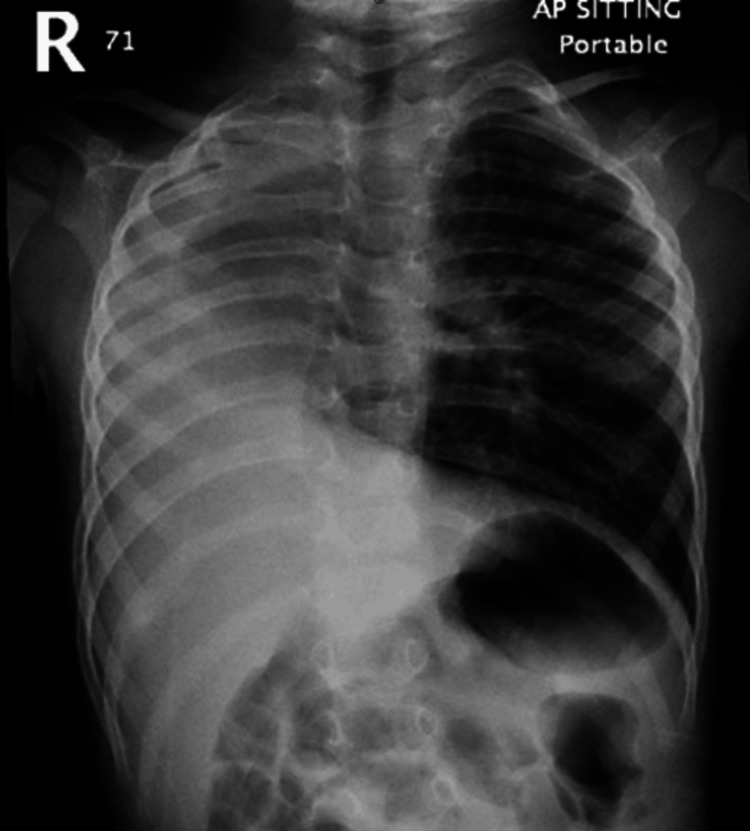
Chest X-ray showing dextrocardia and partial opacification of the right hemithorax and hyperinflation of the left lung

A 22-month-old male, born full-term through spontaneous vaginal delivery, was diagnosed with severe scimitar syndrome since birth, manifesting as dextrocardia and severe hypoplasia of the right pulmonary artery and the right lung. However, no surgical intervention was performed. Additionally, he was diagnosed with bronchial asthma. However, compliance with the prescribed salbutamol and budesonide was poor. He presented to our hospital with a three-day history of cough, rhinorrhea, shortness of breath, vomiting, and decreased oral intake. His symptoms had started after contact with a positive case of upper respiratory tract infection. Upon presentation, he was in respiratory distress, with a respiratory rate reaching 52 breaths per minute, and SpO_2_ ranging between 75 and 80% on room air. Physical examination revealed throat congestion along with mild suprasternal retractions and diminished air entry into the right lung with bilateral wheeze and fine crackles. Moreover, the chest radiograph demonstrated characteristics of scimitar syndrome: dextrocardia and right lung hypoplasia. The left lung was hyperinflated, and a stable right-sided mediastinal shift was noted on previous radiographs, with partial opacification of the right hemithorax (Figure 1).

**Figure 2:**
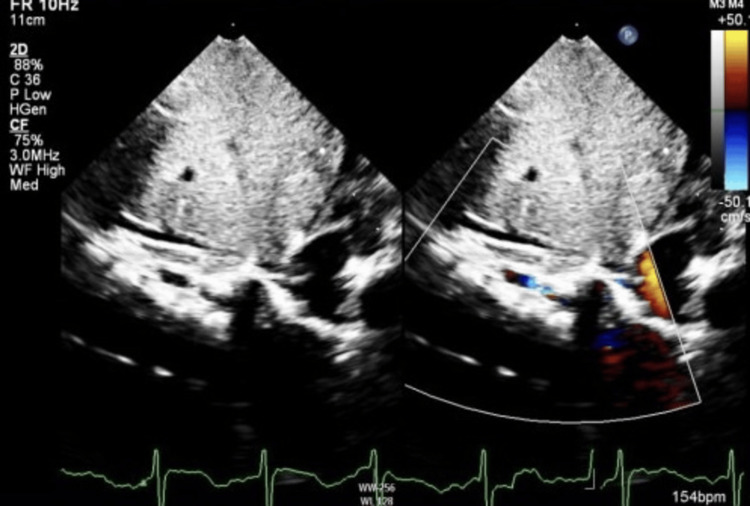
Subcostal view of transthoracic echocardiography with two-dimensional color Doppler demonstrating the anomalous right pulmonary vein drainage into the IVC IVC: inferior vena cava

The patient was subsequently admitted and managed as a case of asthma exacerbation secondary to viral infection; the viral multiplex came back positive for rhinovirus. He was moved to the high-dependency unit (HDU) for close monitoring and observation. His echo showed dextrocardia and abdominal situs solitus. All of the cardiac mass noted was in the right hemothorax. Moreover, there was severe hypoplasia of the right pulmonary artery with absent pulmonary veins and dilated left pulmonary artery. Aortopulmonary collaterals were noted reaching the right lung. The atria and ventricular septum were intact with normal ventricular function and no evidence of pulmonary hypertension, mitral or tricuspid valve regurgitation, left ventricular outflow or right ventricular outflow obstruction, or pericardial effusion (Figure 2).

The patient responded well to asthma management protocol and was discharged home vitally stable and sating well on room air. Upon discharge, he was prescribed fluticasone, montelukast, and salbutamol. The mother was educated about the importance of compliance with prophylactic medications and given a follow-up appointment with pulmonology clinics. This had been the patient's ninth ER visit in less than two years, where he presented with recurrent viral infections triggering asthma exacerbations responsive to asthma protocol. Despite multiple appointments with pulmonology clinics, compliance is still suboptimal.

## Discussion

The severity of scimitar syndrome varies with the age of presentation; the infantile form is associated with a poorer prognosis, and it usually presents soon after birth with pulmonary hypertension, respiratory distress, cyanosis, hemoptysis, and cardiac failure. However, when diagnosed in childhood or adulthood, it is often an incidental finding in an asymptomatic individual or could accompany complaints such as recurrent respiratory tract infection and exertional dyspnea [5]. The diagnosis is often radiological, with the unique scimitar appearance in a chest X-ray where a shadow of the descending pulmonary vein can be observed along the right cardiac border, with dextrocardia and a hypoplastic right lung. The presence of the scimitar sign makes the diagnosis of this illness easier. However, if cardiac shadow obscures the scimitar vein, the diagnosis can still be confirmed by one or more conventional techniques, such as echocardiography, CT, or angiography [6].

Apart from congenital heart defects, scimitar syndrome is also associated with ipsilateral diaphragmatic anomalies, horseshoe lung, localized bronchiectasis, pulmonary lobar and respiratory tract abnormalities, as well as sequestration of the right lower lobe. The Qp/Qs, pulmonary artery pressures, and pulmonary vascular resistance are predicted to rise as a result of these findings, potentially aggravating clinical signs and symptoms such as recurrent pulmonary infections [7]. The hypoplastic lung syndromes, including the variants of scimitar syndrome, offer a rare chance to understand how anomalies in the bronchial tree and its blood drainage system may increase the risk of recurrent respiratory infections.

Earlier authors have noted higher rates of asthma symptoms and abnormalities in pulmonary function in children with ASDs, which accompany the syndrome in 80% of cases [8-9]. However, there is a general dearth of data in the literature about the association between asthma and scimitar syndrome. One of the challenges facing the diagnosis of scimitar syndrome is that it could be mistaken for asthma, as seen in a case report of a four-year-old girl who presented with a chronic cough. Asthma was the main differential, and she received salbutamol without significant improvement, only to be diagnosed later based on chest X-ray findings [10].

There is still uncertainty about the management of this condition, including the kind, timing, and indications of intervention. Medical treatment is indicated during infancy to counteract heart failure and allow growth before surgical repair. If pulmonary hypertension develops, treatment is necessary before surgery, either with medications or by coil embolization to the aortopulmonary collateral arteries to decrease the pulmonary blood flow [8]. Diuretics and Na+/K+ ATPase inhibitors are typically used to treat cardiac symptoms. Antibiotics, corticosteroids, beta-agonists, and ipratropium bromide may aid in the management of respiratory symptoms [2]. Generally, surgical correction is advised for all symptomatic patients as well as asymptomatic patients with pulmonary-to-systemic flow ratios greater than 1.5:1, or pulmonary-to-systemic flow ratios less than 1.5:1, in the context of clinically treated pulmonary hypertension, scimitar vein stenosis, or accompanying cardiac lesions [11]. Depending on the anatomical and pathologic characteristics of each case, the surgical strategy for treating scimitar syndrome varies. Surgical intervention can be achieved through either resection of the lung drained by the anomalous scimitar vein or by corrective rerouting of the flow. Lobectomy is indicated when the patient experiences recurrent respiratory tract infections, persistent hemoptysis, or bronchiectasis [2].

## Conclusions

Scimitar syndrome is a rare condition. A suspicion accompanied by a simple chest radiograph is the appropriate first step toward a diagnosis. Echocardiography and other cutting-edge imaging techniques should be utilized to test for potential related diseases. Patients with scimitar syndrome are typically at higher risk of recurrent respiratory tract infections, which may have aggravated other respiratory conditions such as bronchial asthma in our patient, leading to multiple hospitalizations that could have been avoided with better adherence to prophylactic asthma medications. Hence, a multidisciplinary approach should be applied in patient care.
